# Antifungal Activity of Minocycline and Azoles Against Fluconazole-Resistant *Candida* Species

**DOI:** 10.3389/fmicb.2021.649026

**Published:** 2021-05-13

**Authors:** Jingwen Tan, Shaojie Jiang, Lihua Tan, Haiyan Shi, Lianjuan Yang, Yi Sun, Xiuli Wang

**Affiliations:** ^1^Department of Medical Mycology, Shanghai Skin Disease Hospital, Tongji University School of Medicine, Shanghai, China; ^2^Department of Gastroenterology, Jingzhou Central Hospital, The Second Clinical Medical College, Yangtze University, Jingzhou, China; ^3^Department of Dermatology, Jingzhou Central Hospital, The Second Clinical Medical College, Yangtze University, Jingzhou, China; ^4^Institute of Photomedicine, Shanghai Skin Disease Hospital, Tongji University School of Medicine, Shanghai, China

**Keywords:** minocycline, fluconazole resistant *Candida* spp., *Candida auris*, antifungal, azole, synergy

## Abstract

*Candida* species are the most common fungal pathogens to infect humans, and can cause life-threatening illnesses in individuals with compromised immune systems. Fluconazole (FLU) is the most frequently administered antifungal drug, but its therapeutic efficacy has been limited by the emergence of drug-resistant strains. When co-administered with minocycline (MIN), FLU can synergistically treat clinical *Candida albicans* isolates *in vitro* and *in vivo*. However, there have been few reports regarding the synergistic efficacy of MIN and azoles when used to treat FLU-resistant *Candida* species, including *Candida auris*. Herein, we conducted a microdilution assay wherein we found that MIN and posaconazole (POS) showed the best *in vitro* synergy effect, functioning against 94% (29/31) of tested strains, whereas combinations of MIN+itraconazole (ITC), MIN+voriconazole (VOR), and MIN+VOR exhibited synergistic activity against 84 (26/31), 65 (20/31), and 45% (14/31) of tested strains, respectively. No antagonistic activity was observed for any of these combinations. *In vivo* experiments were conducted in *Galleria mellonella*, revealing that combination treatment with MIN and azoles improved survival rates of larvae infected with FLU-resistant *Candida*. Together, these results highlight MIN as a promising synergistic compound that can be used to improve the efficacy of azoles in the treatment of FLU-resistant *Candida* infections.

## Introduction

Invasive fungal infections represent an increasingly common threat to human health (Firacative, 2020), with *Candida* species serving as the leading cause of fungal infections and the fourth most prominent source of bloodstream infections globally, resulting in over 750,000 infections and a 40% mortality rate globally each year (McCarty and Pappas, 2016; Tsay et al., 2020). *Candida albicans* is the leading pathogenic member of this family, accounting for roughly half of these infections, followed by *Candida glabrata*, *Candida tropicalis*, *Candida parapsilosis*, and *Candida krusei*. In total, these five species cause 90% of candidaemia and other forms of invasive candidiasis (Goemaere et al., 2018).

Fluconazole (FLU) is the most commonly prescribed antifungal drug, but its utility is increasingly limited by the emergence of drug-resistant strains (Perfect and Ghannoum, 2020). Approximately 0.5–2% of *C. albicans* isolates are resistant to FLU, while these resistance frequencies, respectively, range from 4 to 9, 2 to 6, and 11 to 13% for *C. tropicalis*, *C. parapsilosis*, and *C. glabrata* (Berkow and Lockhart, 2017). *Candida auris* is an emerging pathogen, and 93% of these isolates are resistant to FLU with varying levels of resistance to other azoles, making it a particularly dangerous nosocomial pathogen with mortality rates of 30–60% (Berkow and Lockhart, 2017; Forsberg et al., 2019). As such, there is a clear need to identify reliable antifungal agents or compounds capable of enhancing the antifungal activity of extant compounds in order to improve patient outcomes.

Minocycline (MIN) is a second-generation semi-synthetic tetracycline analog that is widely used in clinical settings (Asadi et al., 2020). It exhibits a high degree of fat solubility, and can readily pass through the blood-brain barrier (Yong et al., 2004). MIN exhibits broad-spectrum antibacterial activity, and can be used to combat multidrug-resistant Gram-positive and Gram-negative bacteria (Sapadin and Fleischmajer, 2006). Importantly, MIN has also been shown to function synergistically with FLU when treating clinical *C. albicans* isolates *in vitro* and *in vivo* (Shi et al., 2010; Gu et al., 2018). As such, MIN may represent a promising drug that can be administered in combination with other azoles to treat infections caused by *C. auris* and other pathogenic *Candida* species.

In order to test this hypothesis, we explored the *in vitro* activity of MIN alone or in combination with FLU, itraconazole (ITC), voriconazole (VOR), or posaconazole (POS) against FLU-resistant *Candida* isolates. The *in vivo* effect of drug combination was evaluated using *Galleria mellonella*, as it is an ideal model system for studies of antifungal drug activity.

## Materials and Methods

### Fungal Isolates

In total, 31 *Candida* isolates were utilized in the present analysis, including eight FLU resistant *C. albicans*, three FLU resistant *C. parapsilosis*, four FLU resistant *C. tropicalis*, six FLU susceptible dose-dependent *C. glabrata* strains, and 10 *C. auris* strains. All of these strains were clinical isolates, with the *C. auris* strains having been obtained from the CDC and FDA Antibiotic Resistance Isolate Bank. The identities of all strains were confirmed based on morphological evaluation and sequencing of the ITS and D1/D2 regions. *Candida parapsilosis* (ATCC22019) was included to ensure quality control.

### Antifungal Agents

FLU (No. S1131), VOR (No. S1442), ITC (No. S2476), POS (No. S1257), and MIN (No. S4226) were obtained in a powdered form from Selleck Chemicals (TX, United States), and were prepared as detailed in M27-A4 (CLSI, 2017). Working concentration ranges were 0.03–16 μg/ml for ITC, VOR, and POS, and 0.25–64 μg/ml for FLU and MIN.

### Inoculum Preparation

Yeast conidia were collected from isolates incubated for 24 h on potato dextrose agar (PDA) at 30°C. Yeast conidia were resuspended at 1–5 × 10^6^ cfu/ml in sterile saline, and were then diluted 1,000-fold using RPMI-1640 to yield a suspension that was twice as concentrated as required (1–5 × 10^3^ cfu/ml).

### Testing the *in vitro* Synergy of MIN and Azoles

A broth microdilution checkerboard assay approach was used for the present study, having been adapted from the Clinical and Laboratory Standards Institute (CLSI) M27-A4 (CLSI, 2017). First, 50 μl volumes of MIN serial dilutions were applied horizontally to the wells of a 96-well plate containing 100 μl of prepared inoculum suspension, after which 50 μl volumes of azole serial dilutions were applied vertically to the wells of this same plate. Results were then analyzed after a 24 h incubation at 35°C.

Minimum inhibitory concentration (MIC) values were the lowest drug concentrations that suppressed fungal growth by 50% relative to control treatment at the end of the 24 h incubation. The fractional inhibitory concentration index (FICI) was used to assess MIN and azole interactions, and was calculated with the equation: FICI = (Ac/Aa) + (Bc/Ba), where Ac and Bc are the MIC values of tested agents in combination, while Aa and Ba correspond to these values for single-agent A and B treatments. A FICI of ≤0.5 is considered to indicate synergy, while a FICI of >0.5 to ≤4 is indicative of a lack of any interaction, and a FICI of >4 corresponds to an antagonistic interaction. Experiments were conducted in triplicate.

### Assessment of the *in vivo* Activity of MIN Alone and in Combination With Azoles

As discussed previously (Jiang et al., 2020), survival tests were conducted with sixth instar larvae (300 mg; Sichuan, China) to evaluate the efficacy of MIN as a single-agent and in combination with azole drugs on *G. mellonella* infected with *C. albicans* R14, *C. parapsilosis* N101, *C. tropicalis* 00279, *C. glabrata* 05448, and *C. auris* AR385. Larvae were stored in the dark at room temperature with shavings prior to experimental use, while *Candida* strains had been grown for 2 days on PDA, after which the colony surface was scraped with a sterile plastic loop, washed two times, and adjusted to 1 × 10^8^ cfu/ml using sterile saline. Control larval groups injected with saline, conidial suspensions, or nothing were established.

To explore the *in vivo* synergistic activity of MIN and azoles against pathogenic fungi, nine treatment groups were established: MIN, FLU, ITC, POS, VOR, MIN+FLU, MIN+ITC, MIN+POS, and MIN+VOR groups. Conidia suspensions were inoculated into larvae (10 μl per larvae) using a Hamilton syringe (25 gauge, 50 μl), and antifungal agents or a control solution (1 μg per larvae; drug concentration = 200 mg/L) was introduced into the larvae through the last left proleg after the area was cleaned with an alcohol swab. Within 120 h following infection, larval survival rates were recorded every 24 h. *Galleria mellonella* survival curves were assessed *via* the Kaplan-Meier method and the log-rank (Mantel-Cox) test, with *p* < 0.05 as a significance threshold.

## Results

### MIN and Azoles Interactions *in vitro*

A checkerboard microdilution assay was initially performed to explore the antifungal activity levels of different azoles alone and in combination with MIN when used to treat different *Candida* spp. *in vitro* (Tables 1 and 2). POS showed the best synergistic activity with MIN, achieving activity against 100% of tested *C. albicans*, *C. parapsilosis*, *C. tropicalis*, and *C. glabrata* strains and against 80% of tested *C. auris* strains. A combination of MIN and ITC exhibited synergistic activity against 100% of *C. parapsilosis* strains, 90% of *C. auris* strains, 83% of *C. glabrata* strains, and 75% of *C. albicans* and *C. tropicalis* strains. In combination with FLU, MIN exhibited synergistic efficacy against 75% of *C. albicans*, 70% of *C. auris*, 67% *C. glabrata*, 50% *C. tropicalis*, and 33% *C. parapsilosis* strains. Combination MIN and VOR treatment exhibited the poorest synergistic activity, affecting just 80% of *C. auris*, 50% of *C. albicans*, 33% of *C. glabrata*, and 0% of *C. parapsilosis* and *C. tropicalis* strains.

**Table 1:** Minimum inhibitory concentration (MIC) values pertaining to combinations of minocycline (MIN) and azoles when used to treat *Candida* spp.

No.	Species	MICs (μg/ml)^1^								
		Agent alone					Combination^2^			
		MIN	ITC	VOR	POS	FLU	MIN/ITC	MIN/VOR	MIN/POS	MIN/FLU
ATCC 64550	*C.albicans*	>64	1	0.5	0.5	8	16/0.5(I)	1/0.25(I)	16/0.125(S)	1/8(I)
R1		>64	8	2	2	16	4/1(S)	16/0.5(S)	4/0.5(S)	4/2(S)
R3		>64	2	1	2	32	4/1(I)	32/0.125(I)	4/0.25(S)	1/16(I)
R4		>64	4	1	2	16	8/0.5(S)	32/0.5(I)	4/0.25(S)	16/4(S)
R9		>64	>16	16	8	>64	8/0.5(S)	8/0.25(S)	4/0.125(S)	2/1(S)
R14		>64	4	8	2	16	2/0.5(S)	2/0.5(S)	2/0.25(S)	1/4(S)
R15		>64	2	2	1	32	4/0.5(S)	4/0.25(S)	4/0.125(S)	8/4(S)
N175		>64	4	1	1	8	4/0.5(S)	2/0.5(I)	4/0.25(S)	16/0.5(S)
N87	*C.parapsilosis*	>64	4	0.5	1	16	4/0.5(S)	32/0.5(I)	4/0.125(S)	16/4(S)
N101		>64	4	1	1	>32	8/1(S)	32/0.125(I)	4/0.125(S)	32/1(I)
N112		>64	4	1	0.5	16	8/0.5(S)	8/0.5(I)	4/0.125(S)	32/1(I)
00279	*C.tropicalis*	>64	1	0.5	0.5	64	16/0.5(S)	1/0.5(I)	8/0.125(S)	32/64(I)
N205		>64	4	0.5	1	32	32/1(I)	32/0.5(I)	8/0.25(S)	8/2(S)
N331		>64	2	0.5	1	32	4/0.5(S)	32/0.25(I)	8/0.25(S)	32/1(I)
N336		>64	4	1	1	32	8/0.5(S)	32/1(I)	4/0.25(S)	16/4(S)
05448	*C. glabrata*	64	4	0.5	2	8	8/0.5(S)	4/0.125(S)	8/0.125(S)	16/0.5(S)
C5		>64	1	0.5	1	8	2/0.5(I)	32/0.125(I)	8/0.125(S)	16/1(S)
C35		>64	2	0.5	1	8	8/0.5(S)	32/0.5(I)	4/0.25(S)	16/2(S)
C128		>64	1	2	1	8	8/0.25(S)	4/0.5(S)	8/0.25(S)	16/1(S)
N180		>64	2	1	1	8	2/0.5(S)	32/0.125(I)	4/0.125(S)	4/4(I)
N199		>64	4	1	1	>32	8/0.5(S)	32/0.5(I)	2/0.25(S)	16/16(I)
AR381	*C. auris*	>64	0.125	0.125	0.125	4	1/0.125(I)	1/0.125(I)	1/0.125(I)	16/2(I)
AR382		>64	0.5	1	0.5	16	8/0.125(S)	2/0.25(S)	1/0.125(S)	8/1(S)
AR383		>64	1	4	0.25	128	4/0.25(S)	4/1(S)	1/0.125(I)	8/8(S)
AR384		>64	2	0.5	0.5	128	8/0.125(S)	2/0.125(S)	8/0.125(S)	16/2(S)
AR385		>64	1	8	1	128	8/0.25(S)	1/1(S)	4/0.125(S)	16/4(S)
AR386		>64	1	16	0.5	128	4/0.125(S)	1/1(S)	4/0.125(S)	32/8(I)
AR387		>64	1	1	0.5	8	2/0.125(S)	2/0.125(S)	2/0.125(S)	4/1(S)
AR388		>64	2	4	0.5	128	8/0.5(S)	16/4(I)	8/0.125(S)	16/4(S)
AR389		>64	1	4	0.5	128	16/0.25(S)	16/1(S)	8/0.125(S)	8/2(S)
AR390		>64	0.5	4	0.5	128	8/0.125(S)	4/0.125(S)	8/0.125(S)	32/8(I)

1 The MIC is the concentration resulting in 50% growth inhibition.

2 fractional inhibitory concentration index (FICI) results are shown in parentheses. S, synergy (FICI <0.5); I, no interaction (indifference, 0.5 < FICI < 4).

**Table 2:** Summary of *in vitro* drug interactions.

Species(*n*)	*n* (%) of isolates showing synergism for the combination			
	MIN/ITC	MIN/VOR	MIN/POS	MIN/FLU
*C.albicans* (8)	6(75%)	4(50%)	8(100%)	6(75%)
*C.parapsilosis* (3)	3(100%)	0(0%)	3(100%)	1(33%)
*C.tropicalis* (4)	3(75%)	0(0%)	4(100%)	2(50%)
*C.glabrata* (6)	5(83%)	2(33%)	6(100%)	4(67%)
*C.auris* (10)	9(90%)	8(80%)	8(80%)	7(70%)
Total (31)	26(84%)	14(45%)	29(94%)	20(65%)

### MIN and Azoles Interactions *in vivo*

**Figure 1:**
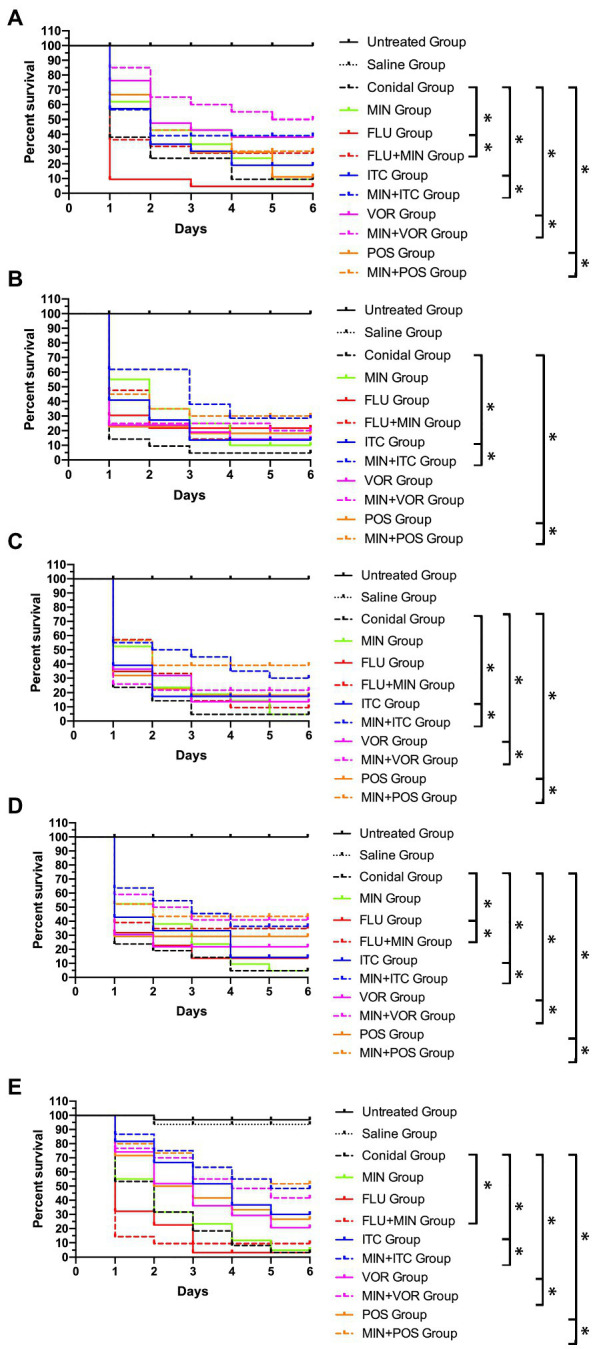
*Galleria mellonella* survival curves following infection with *Candida* spp. **(A)** *Candida albicans* R14; **(B)** *Candida parapsilosis* N101; **(C)** *Candida tropicalis* 00279; **(D)** *Candida glabrata* 05448; **(E)** *Candida auris* AR385; Untreated Group, wild type uninfected larvae; Saline Group, wild type larvae injected with saline; Conidial Group, larvae infected with *Candida* without any treatment; MIN Group, *Candida*-infected larvae treated with MIN only; fluconazole (FLU) Group, *Candida*-infected larvae treated with FLU only; itraconazole (ITC) Group, *Candida*-infected larvae treated with ITC only; voriconazole (VOR) Group, *Candida*-infected larvae treated with VOR only; posaconazole (POS) Group, *Candida*-infected larvae treated with POS only; MIN+FLU Group, *Candida*-infected larvae treated with MIN combined with FLU; MIN+ITC Group, *Candida*-infected larvae treated with MIN combined with ITC; MIN+VOR Group, *Candida*-infected larvae treated with MIN combined with VOR; MIN+POS Group, *Candida*-infected larvae treated with MIN combined with POS. ^∗^*p* < 0.05.

Next, we performed *in vivo* antifungal activity assays using *G. mellonella* as a model system, with survival rates for the larvae in each group being shown in Table 3 and Figure 1. Treatment with MIN alone had no effect on any of the five *Candida* spp. groups. When combined with FLU, however, this treatment significantly prolonged the survival of larvae infected with *C. albicans*, *C. glabrata*, and *C. auris* (*p* < 0.05), with a particularly noteworthy increase in the survival rate of larvae infected with *C. glabrata* from 5 to 25%. Combination treatment with ITC was associated with significantly prolonged larval survival rates in all groups (*p* < 0.05), with a particularly pronounced increase of 30% in the *C. glabrata* group. Combination MIN + VOR treatment significantly prolonged the survival (*p* < 0.05) of all larvae other than those infected with *C. parapsilosis*, with maximal synergy being observed for larvae infected with *C. tropicalis* for which the survival rate rose by 30%. Combination MIN + POS treatment also significantly (*p* < 0.05) increased survival in all groups, particularly in the *C. auris* group in which the survival rate reached 51.67%.

**Table 3:** Summary of *in vivo* drug interactions.

Drugs	Survival rate				
	*C. abicans*	*C. parapsilosis*	*C. tropicalis*	*C. glabrata*	*C. auris*
FLU	0.00%	10.00%	5.00%	5.00%	0.00%
FLU+MIN	20.00%	5.00%	5.00%	25.00%	5.00%
ITC	15.00%	5.00%	5.00%	10.00%	30.00%
ITC+MIN	30.00%	25.00%	30.00%	40.00%	48.33%
VOR	35.00%	10.00%	5.00%	10.00%	20.00%
VOR+MIN	50.00%	20.00%	35.00%	25.00%	41.67%
POS	10.00%	10.00%	10.00%	15.00%	26.67%
POS+MIN	25.00%	30.00%	30.00%	35.00%	51.67%

## Discussion

Minocycline was first identified as an inhibitor of *C. albicans* growth in 1974 (Waterworth, 1974), and several studies have further highlighted the antifungal activity of this compound. Shi et al. (2010) and Gu et al. (2018) demonstrated the ability of MIN to synergize with FLU against *C. albicans in vitro* and *in vivo* and Gao et al. (2013) observed synergy between tetracycline and FLU when treating *C. albicans* biofilms. MIN has also been shown to synergize with azoles in the treatment of other fungal species. Kong et al. (2020) determined that MIN was able to synergize with FLU *in vivo* and *in vitro* when treating *Cryptococcus neoformans*, while Gao et al. (2020) reported synergy between MIN and azoles when treating clinically important *Aspergillus*, *Fusarium*, and *Exophiala dermatitidis* isolates. Herein, we explored the synergistic activity of MIN in combination with azoles when treating FLU-resistant *C. albicans*, *C. parapsilosis*, *C. tropicalis*, *C. glabrata*, and *C. auris*.

Consistent with other studies, we found that MIN was able to enhance fungal sensitivity to azole treatment both *in vitro* and *in vivo*. In our *in vitro* analyses, MIN and POS showed 100% *in vitro* synergy effect in *C. albicans*, *C. parapsilosis*, *C. tropicalis*, *C. glabrata* group, functioning against 94% (29/31) of tested strains, whereas combinations of MIN+ITC, MIN+FLU, and MIN+VOR exhibited synergistic activity against 84 (26/31), 65 (20/31), and 45% (14/31) of tested strains, respectively. The *C. auris* strains used in this study belonged to four different clusters, with AR382, AR387, AR388, AR389, and AR390 belonging in cluster I, AR381 belonging in cluster II, AR383 and AR384 belonging in cluster III, and AR385 and AR386 belonging in cluster IV (Vatanshenassan et al., 2020). No differences among these clusters were detected in our *in vitro* analyses.

Our *in vivo* experiment utilized *G. mellonella* as an animal model, given that these larvae exhibit similar responses to those of mammals and can be used as an ideal model system for studies of antifungal drug activity. Our data indicated that MIN and VOR combination treatment exhibited the best synergistic efficacy in the context of *C. albicans* and *C. tropicalis* infections, while MIN+POS was most effective against *C. parapsilosis* and *C. auris*. For *C. glabrata*, a combination of MIN+ITC was most effective. However, our *in vitro* data were not in full accordance with our *in vivo* data, potentially because too few strains were used when conducting our *in vivo* study. Additionally, further studies that using mice as infection models are required.

In our data, MIN can reduce the MIC of azoles in some tested strains, while there was no change for the others. That can observed in others study either, Shi et al. (2010) showed MIN can reduce MIC of FLU in 50% tested *C. albicans* strains. We speculate that might associate with the mechanism of synergistic action. Though remains incompletely understood, the ability of MIN to enhance FLU efficacy may be related to efflux pump blockade, the stimulation of high levels of intracellular calcium, iron chelation, or the inhibition of mitochondrial functionality (Oliver et al., 2008; Shi et al., 2010; Fiori and Van Dijck, 2012; Gao et al., 2013). Further research that can clarify the mechanisms might help to address this question.

In summary, we found that MIN can synergize with azoles to improve the antifungal activity of these agents against azole-resistant *Candida* species, with particular efficacy against *C. auris in vitro* and *in vivo*. Given that resistance to azoles is associated with significant increases in treatment failure and mortality rates, overcoming drug resistance is a critical public health issue. Our results highlight the potential of MIN as a tool that can overcome such azole resistance when used to treat *Candida* infections, although further work will be required to confirm these results and to elucidate the underlying mechanisms. Even so, our results hold great promise, suggesting that MIN can be used to reliably enhance efforts to cure invasive azole-resistant *Candida* infections in clinical settings.

## Data Availability Statement

The original contributions presented in the study are included in the article/supplementary material; further inquiries can be directed to the corresponding authors.

## Author Contributions

JT, SJ, and LT carried out the *in vitro* and *in vivo* antifungal experiment. JT and HS collected and analyzed the experiment data. YS and XW designed and interpreted the experiment data and wrote the manuscript. LY and XW revised the manuscript critically for important content. All authors contributed to the article and approved the submitted version.

### Conflict of Interest

The authors declare that the research was conducted in the absence of any commercial or financial relationships that could be construed as a potential conflict of interest.
